# Pleomorphic Xanthoastrocytoma of the Pineal Region in a Pediatric Patient With Neurofibromatosis Type 1

**DOI:** 10.31486/toj.18.0156

**Published:** 2020

**Authors:** Joshua A. Hanna, Mansour Mathkour, Edna E. Gouveia, JonMark Lane, Lauren Boehm, Joseph R. Keen, Erin E. Biro, Olawale A. Sulaiman, Cuong J. Bui

**Affiliations:** ^1^ Department of Neurosurgery, Ochsner Clinic Foundation, New Orleans, LA; ^2^ Department of Neurosurgery, Tulane Medical Center, New Orleans, LA; ^3^ The University of Queensland Faculty of Medicine, Ochsner Clinical School, New Orleans, LA

**Keywords:** *Neurofibromatosis type 1*, *pediatrics*, *pineal region*, *pleomorphic astrocytoma*

## Abstract

**Background:** Pleomorphic xanthoastrocytoma (PXA) is a rare and often focal glioma that most commonly affects children and young adults. Lesions are preferentially supratentorial and superficial, although infratentorial masses have been described, along with occasional involvement of the leptomeninges. The World Health Organization (WHO) categorizes these tumors as grade II, with surgical resection carrying a favorable prognosis. However, these tumors may undergo malignant degeneration and must be identified for appropriate treatment and prognosis. PXA has been associated with neurofibromatosis type 1 (NF1), although it is not the classic phenotype of NF1. We present a novel report of PXA, atypically located in the pineal region of a patient with a history of NF1.

**Case Report:** A 17-year-old male with a history of NF1 presented with 1 month of bifrontal headaches. Magnetic resonance imaging was significant for a heterogeneous tectal mass, suspicious for a glioma extending to the fourth ventricle and causing displacement of the cerebral aqueduct without obstructive hydrocephalus. Following an infratentorial-supracerebellar approach for tumor resection, histopathology confirmed a low-grade variable neoplasm consistent with PXA. Postoperative imaging confirmed gross total resection with no evidence of recurrence at 9 months postoperatively.

**Conclusion:** To our knowledge, this case is the fifth report of pineal PXA and the first associated with NF1. Because PXA presents similarly to other NF1-related intracranial tumors, careful diagnosis via immunohistochemistry is imperative. Gross tumor resection is usually curative; however, PXA has the propensity to undergo malignant degeneration and may require adjuvant treatment.

## INTRODUCTION

Pleomorphic xanthoastrocytoma (PXA) is a rare brain neoplasm that occurs most often in the cerebral hemispheres of young adults and children. PXA was first reported by Kepes et al,^1^ and because of its proclivity for the temporal lobes, commonly presents with seizures. Outside the hemispheres, PXAs have been found in the cerebellum, retina,^2^ basal ganglia, thalamus, and spinal cord^3^ but are exceedingly rare in the pineal region. Srinivas et al^4^ described the first pineal region PXA in 2010, and only 4 cases have been reported in the literature. Our case represents a confluence of rarities in that we report what we believe to be the fifth case of PXA in the pineal region and the first in a patient with neurofibromatosis type 1 (NF1).

NF1 is an autosomal dominant genetic disorder with complete penetrance involving the chromosomal 17q11.2 gene locus.^5^ NF1 is most commonly associated with pilocytic astrocytomas in the optic apparatus and brainstem. Occasionally, these neoplasms are found to be diffuse astrocytoma or glioblastoma. NF1 is rarely associated with PXA, with a total of 16 cases reported since 1993 (Table 1).^6-19^ These PXAs most often involved the supratentorial cerebral cortex; however, in 3 cases, they were in the cerebellum, and in 2 cases, the PXAs were in the basal ganglia and ventricular-thalamic area. The natural history and prognosis of PXAs in atypical locations and in association with NF1 are unknown. Because PXAs are World Health Organization (WHO)^20^ grade II neoplasms with a propensity to undergo malignant degeneration, careful immunohistochemical analysis is imperative to help differentiate them from other more common tumors associated with NF1 and thus guide treatment, prognostication, and surveillance.

**Table 1.** Neurofibromatosis Type 1–Associated Pleomorphic Xanthoastrocytomas

Case	Age, Sex	Location	Classification
Ozek et al, 1993^6^	14, M	Medial temporal	Benign
Kubo et al, 1996^7^	21, F	Parietal cortical	Benign
Koeller and Henry, 2001^8^	13, M	Frontal, cortical	Unknown
Naidich et al, 2004^9^	51, F	Cerebellar, vermian	Benign
Saikali et al, 2005^10^	36, F	Occipital deep, cerebellar cortical	Anaplastic
Hariharan et al, 2006^11^	39, F	Frontal deep	Anaplastic
Horiguchi et al, 2011^12^	32, M	Basal ganglia	Benign
Adeleye et al, 2012^13^	10, M	Ventricular, thalamic	Benign
Neal et al, 2012^14^	23, M	Parietal	Benign
Neal et al, 2012^14^	28, M	Occipital	Benign
Prayson, 2012^15^	38, F	Temporal, occipital	Benign
Vizcaino et al, 2014^16^	20, F	Left frontal	Anaplastic
Takei et al, 2015^17^	33, F	Cerebellar	Benign
Thara et al, 2017^18^	42, M	Temporal, parietal	Anaplastic
Singla et al, 2018^19^	25, M	Frontal	Malignant
Yoshihiro, 1998^a^	Unknown	Unknown	Malignant
Our case	17, M	Cerebellar, vermian	Benign

^a^Full text article unavailable in Embase or elsewhere.

We discuss the diagnostic challenges associated with PXA, especially in a patient with NF1.

## CASE REPORT

A 17-year-old male presented with 1 month of mild to moderate headaches and blurry vision. His medical history was significant for NF1, with multiple periorbital tumors that had required surgical resection. Physical examination was benign, and neurologic examination was normal except for bilateral blurry vision.

**Figure 1.**
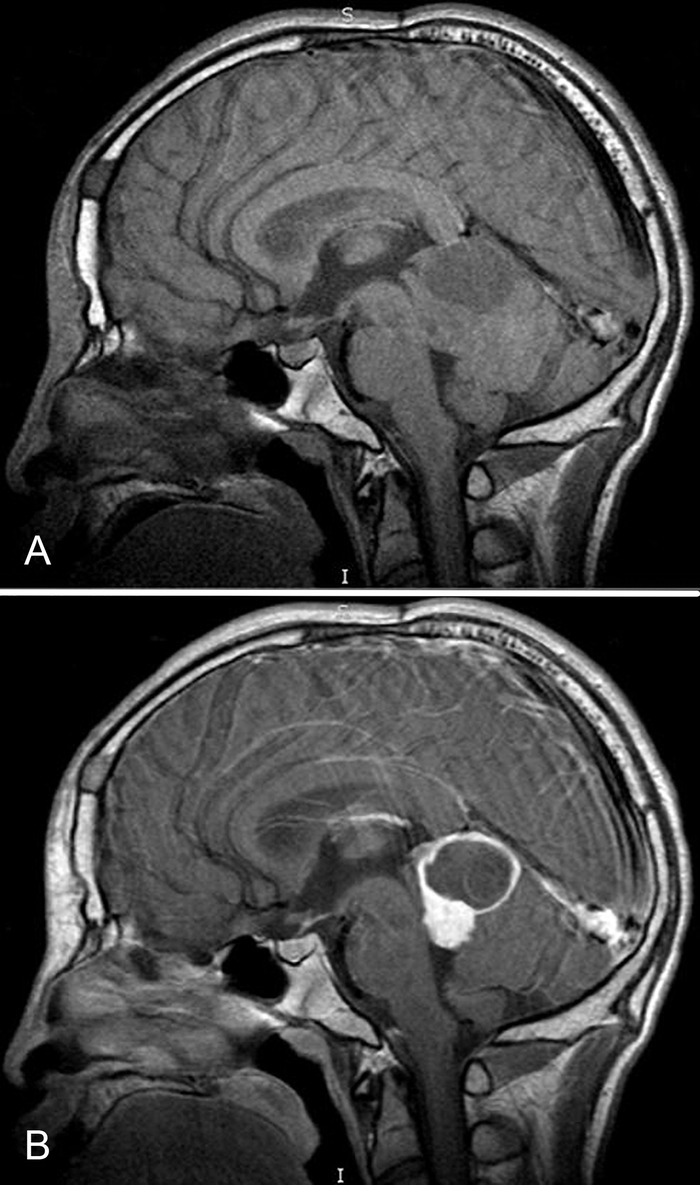
**Magnetic resonance imaging without contrast (A) and with contrast (B) shows a solid and cystic enhancing mass measuring 3.9 cm × 3.6 cm × 2.4 cm centered in the region of the tectum, extending down to the superior aspect of the fourth ventricle with mass effect on the posterior midbrain and displacement of the cerebral aqueduct and with maintained patency.**

Magnetic resonance imaging (MRI) revealed a solid and cystic enhancing mass centered in the region of the tectum that extended inferiorly to the superior aspect of the fourth ventricle (Figure 1). The lesion measured 3.9 cm × 3.6 cm × 2.4 cm and displaced the posterior midbrain and cerebral aqueduct without complete obstruction. There was slight prominence of the lateral ventricles bilaterally, with enlargement of the temporal horns.

The patient underwent a suboccipital infratentorial-supracerebellar approach for tumor resection during which an incision from the inion to C2 was outlined for enough exposure. Intraoperative neuronavigation was used to place 2 burr holes just caudal to the transverse sinus bilaterally, followed by a craniotomy. The tumor was best approached by a dissection over the cerebellar hemispheres and an incision on the cerebellar vermis. Grossly, the tumor was yellow-grey and partially collapsed after decompression of the cystic component. The tumor, which extended inferior to the roof of the fourth ventricle, was removed without difficulty using microsurgical technique and ultrasonic aspiration.

**Figure 2.**
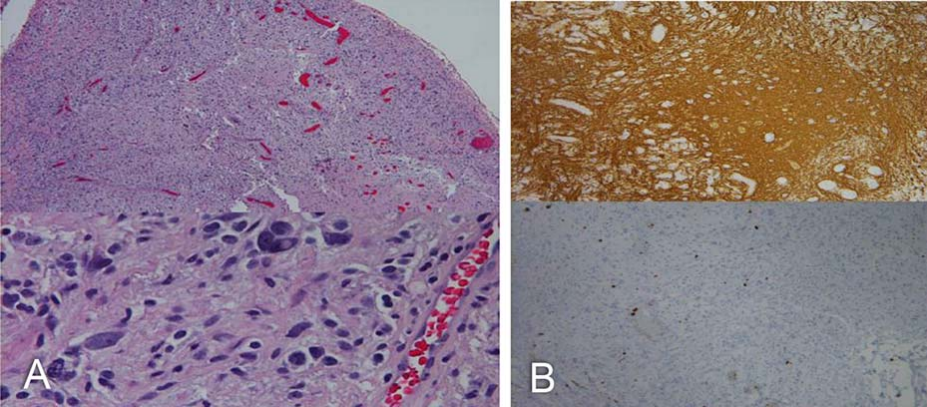
**(A) Hematoxylin and eosin stain of the tumor cells shows marked heterogeneity. In some areas, the tumor cells had round to slightly oval nuclei with mild to moderate nuclear pleomorphism set in a fibrillary background. In other areas, the tumor nuclei were markedly pleomorphic with occasional multinucleated giant cells throughout. (B) Intranuclear cytoplasmic inclusions are prominent throughout, in addition to a high degree of vascularization. All tumor cells were diffusely and strongly positive for glial fibrillary acidic protein (top); Ki-67 (bottom) and reticulin stains showed the presence of pericellular fibers. The Ki-67 proliferation index was between 1% and 2%.**

Pathology was consistent with PXA (Figure 2). Cytologically, the tumor cells showed marked heterogeneity. In some areas, the tumor cell nuclei were round to slightly oval, with mild to moderate nuclear pleomorphism set in a fibrillary background. In other areas, the nuclei were markedly pleomorphic with occasional multinucleated giant cells. Intranuclear cytoplasmic inclusions were prominent throughout, in addition to a high degree of vascularization. All tumor cells were diffusely and strongly positive for glial fibrillary acidic protein (GFAP). Reticulin stains showed the presence of pericellular fibers, and the Ki-67 proliferation index was 1% to 2%.

**Figure 3.**
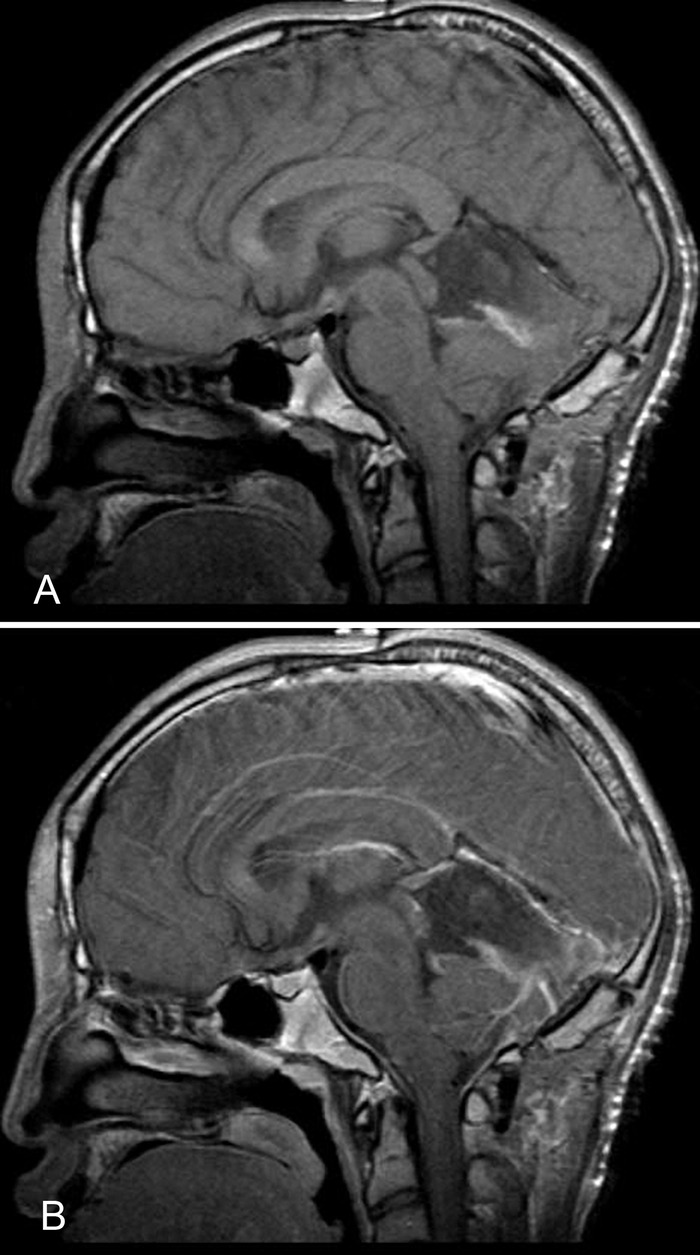
**Magnetic resonance imaging without contrast (A) and with contrast (B) after surgical treatment showed postoperative changes and no evidence of residual tumor.**

Postoperatively, the patient recovered well without any neurologic deficits and was discharged 5 days later. Following discharge, the patient resumed his normal daily activities. Postoperative MRI showed no evidence of residual tumor (Figure 3), and the patient was managed expectantly without the need for adjuvant radiation or chemotherapy. At 4-week, 8-week, 6-month, and 9-month postoperative follow-up, the patient had resolution of symptoms and no evidence of recurrence.

## DISCUSSION

A growing body of literature provides histologic and genetic bases for differentiation of PXA. Such means of differentiation become especially important when PXAs occur in unusual locations, such as the pineal region. In general, tumors of the pineal gland are rare, accounting for 0.4% to 1.0% of all intracranial lesions in adults, 2% to 8% of intracranial neoplasms in children, and <1% of all intracranial tumors overall.^4^ Primary pineal tumors can be categorized as germ cell tumors, which are most common, or as pineal parenchymal tumors, which are comprised of low-grade pineocytomas, intermediate-grade pineal parenchymal tumors, and highly malignant pineoblastoma.^21^ Other neoplasms in the pineal region can arise from cell types in the vicinity of the pineal gland such as meningioma, astrocytic tumors, and metastases from distal sites.^22^ Astrocytic tumors include pilocytic astrocytoma, anaplastic astrocytoma, glioblastoma multiforme, pleomorphic granular cell astrocytoma,^23,24^ and PXA.

The 4 known cases of PXA of the pineal region are listed in Table 2.^4,23,25,26^ Each patient presented with signs and symptoms of increased intracranial pressure, including severe headache and papilledema from obstructive hydrocephalus. The patient with the PXA that underwent anaplastic transformation additionally presented with dementia and gait disturbance.^25^ Our patient's blurred vision and headaches were mild compared to the other cases; because of the heightened vigilance for intracranial tumors in patients with NF1, his diagnosis was made early before he developed obstructive hydrocephalus.

**Table 2.** Pleomorphic Xanthoastrocytomas of the Pineal Region

Case	Age, Sex	Classification	Treatment	Outcome
Srinivas et al, 2010^4^	30, M	Benign	Resection	None reported
Ohta et al, 2010^23^	67, F	Benign	Resection	No recurrence at 24 months
Thakar et al, 2012^26^	15, M	Benign	Resection	None reported
Katayama et al, 2013^25^	61, M	Anaplastic	Partial resection, chemotherapy and radiation	Improvement
Our case	17, M	Benign	Resection	No evidence of recurrence at 9 months

Generally, surgical resection of PXAs is curative; however, malignant or anaplastic characteristics are associated with recurrence and poor outcome and may require radiation, chemotherapy, or other adjuvant therapies.^11,12,19^ Whether an association with NF1 portends a different prognosis for PXAs is uncertain. Of 15 NF1-associated PXAs with a known classification reported in the literature, 4 tumors were anaplastic and 2 were malignant. NF1-associated PXAs, therefore, appear to have worse outcomes than PXAs in the general population, as the malignant or anaplastic transformation in the general population is estimated to be 10% to 20%.^19^ Accurate diagnosis of PXAs in patients with NF1 is essential and should be considered in atypical presentations or locations.

Diagnosing a pineal PXA in the setting of NF1 is challenging, especially considering the plethora of other tumors more commonly associated with NF1. Histologic characteristics include eosinophilic granules, xanthomatous changes, and nuclear and cellular pleomorphism.^27^ Because of their histologic similarity to other tumors, such as those of the pineal region, PXAs are differentiated by immunohistochemical, structural, and genetic markers.^27^ For example, PXAs share a bizarre morphology with the malignant and aggressive giant cell glioblastoma multiforme^28,29^ that may also occur in the pineal region.^14^ Our patient's tumor was notably heterogeneous as it showed both marked and moderate pleomorphism in different areas, a high degree of vascularization, and multinucleated giant cells. Although these characteristics are found in glioblastoma multiforme, that diagnosis was ruled out on the basis of the low Ki-67 index and the absence of mitoses or necrosis. Another diagnostic possibility was pleomorphic granular cell tumor. Both tumors are rarely found in the pineal region, they share a similarly indolent course, and their foundational histology shows lipidized pleomorphic granular cells.^24,25^ However, pleomorphic granular cell tumors show an absence of reticulin fibers and the presence of large numbers of mitochondria,^4^ which is incongruent with our pathologic findings. To further distinguish between the 2 tumor types, PXAs show diffuse GFAP positivity compared to an occasional weakly positive GFAP positivity in pleomorphic granular cell tumors. Our pathologic specimen was diffusely positive for GFAP and stained positive for pericellular reticulin fibers.

Despite advances in immunohistochemical analysis, distinguishing PXA from pilocytic astrocytoma, which is the most common intracranial tumor associated with NF1, can be challenging. Koelsche et al found the presence of a BRAF V600E mutation in 70% of PXAs, principally within the temporal lobe, and proposed a common molecular mechanism of abnormal Raf kinase activity for both NF1-associated lesions and NF1-associated PXAs.^30^ A study by Jacob et al also suggested a possible association between BRAF V600E gene mutations and pilocytic astrocytoma.^31^ In contrast are the 2 reports of BRAF negativity in NF1-associated PXAs.^16,17^ Because of the historic lack of BRAF testing in NF1-associated PXAs, BRAF significance will remain unclear until larger studies are analyzed. In the meantime, morphologic features will continue to be used to distinguish PXAs from pilocytic astrocytomas. BRAF mutation has also been suggested as a marker of progression-free survival in PXAs,^32^ and salvage therapy with the BRAF inhibitor vemurafenib for recurrent PXAs has shown some success.^33^ Although we did not analyze BRAF and other genetic mutations in our case, we advocate for BRAF analysis in all cases of PXA, especially NF1-associated PXAs. Establishing the link between BRAF mutations and PXAs has the potential to provide a more comprehensive understanding of etiology, diagnosis, and treatment.

In a 2019 study of 19 patients with PXA of which 15 underwent anaplastic progression, Phillips et al identified a combination of CDKN2A biallelic deletion and an Raf alteration in all patients in the cohort, suggesting a specific genetic profile, as well as an indicator for anaplastic progression in PXA.^34^ Furthermore, 47% of anaplastic PXAs were found to have a telomerase reverse transcriptase (TERT) gene alteration. This study and others may further elucidate the role of TERT alterations in PXAs, as that role has not been well defined.^35^

## CONCLUSION

PXAs are rare, WHO grade II brain neoplasms that are rarely associated with NF1. Although PXAs are predominantly temporal lobe–based lesions, they have been found in atypical locations such as the pineal region. To our knowledge, we report only the fifth case of a pineal region PXA and the first case in a patient with NF1. Although the literature is scarce, NF1-associated PXAs may portend a higher degree of malignancy and poorer outcomes compared to PXAs that are not associated with NF1; therefore, diagnostic vigilance via histopathologic and immunohistochemical analyses is imperative. Distinguishing PXA from its similar glial counterparts is also important. Genetic mutations may not only help in diagnosis but also potentially help us understand tumor genesis and provide new targets for treatment. PXA should be included in the differential for NF1-associated intracranial lesions, regardless of location.

## Notes

This article meets the Accreditation Council for Graduate Medical Education and the American Board of Medical Specialties Maintenance of Certification competencies for Patient Care and Medical Knowledge.
